# Case report of hepatic retiform hemangioendothelioma: A rare tumor treated with ultrasound-guided microwave ablation

**DOI:** 10.1515/biol-2022-0592

**Published:** 2023-06-14

**Authors:** Tian-Tian Qin, Li-Ting Cao, Li-Xia Lu, Xiu-Li Wang, Jiu-Cong Zhang, Bin Li, Xiao-Hui Yu, Xiao-Long Ren

**Affiliations:** Department of Gastroenterology, The 940 Hospital of Joint Logistic Support Force of People’s Liberation Army, No. 333 of Binhenan Road, Qilihe District, Lanzhou, 730050, China; Department of Infectious Diseases, The Second Affiliated Hospital of Air Force Medical University, Xi’an 710038, China; Department of Ultrasonography, The 940 Hospital of Joint Logistic Support Force of People’s Liberation Army, No. 333 of Binhenan Road, Qilihe District, Lanzhou, 730050, China

**Keywords:** hepatic retiform hemangioendothelioma, microwave ablation, ultrasound, case report

## Abstract

Retiform hemangioendothelioma (RH) is a type of low-grade malignant angiosarcoma. It commonly involves the skin and subcutaneous tissue of the lower extremities, but a few cases have been reported in the gut. However, hepatic RH has not been previously reported. This report presents the case of RH of the liver in a 61-year-old woman who was admitted to the hospital having presented with liver space–occupying lesions of 2 months evolution. The patient underwent an abdominal ultrasound examination, which indicated a hemangioma, but abdominal computed tomography diagnosed a liver abscess. In order to determine the nature of the lesion, an ultrasound-guided liver biopsy was performed, after which a pathological diagnosis confirmed the presence of RH in the liver. The patient underwent ultrasound-guided microwave ablation three times and has been followed up for 8 years with no tumor recurrence or metastasis. Surgical excision is still the first choice for the treatment of hepatic RH. As shown in this case, however, for patients who refuse to undergo surgery or have surgical contraindications, ultrasound-guided microwave ablation is an alternative treatment option. The report of this case expands the scope of liver tumors to a certain extent and provides a reference for clinical diagnosis and treatment.

## 1 Introduction

Retiform hemangioendothelioma (RH) is a rare vascular tumor of low-grade malignancy first described by Calonje in 1994, with less than 40 cases reported throughout the world [1]. Its prognosis is unpredictable because it can affect multiple organs [2]. Unfortunately, a consensus has not yet been reached regarding the effective treatment of this type of tumor.

This article presents the first reported case of hepatic RH. This case is also the first patient with hepatic RH to have been treated successfully with ultrasound-guided microwave ablation.

## 2 Case presentation

A 61-year-old female patient was admitted to our hospital having presented with liver space–occupying lesions of 2 months evolution. The patient underwent a plain abdominal computed tomography (CT) scan and enhanced examination 2 months ago, which identified a round low-density shadow with fuzzy boundaries and uneven density, measuring 43 × 45 mm, in the right lobe of the liver. After contrast enhancement, the edge of the lesion in the arterial phase showed spot-like enhancement, and the contrast medium filled the lesion with time in the venous phase and the delayed phase, which indicated a hemangioma. Due to the non-special clinical manifestations, the patient was given no treatment. Before admission, the patient underwent another abdominal ultrasound examination in a different hospital, which also identified a hemangioma, but an abdominal CT scan diagnosed a liver abscess. The patient was admitted to our hospital for further diagnosis and treatment.

**Figure 1:**
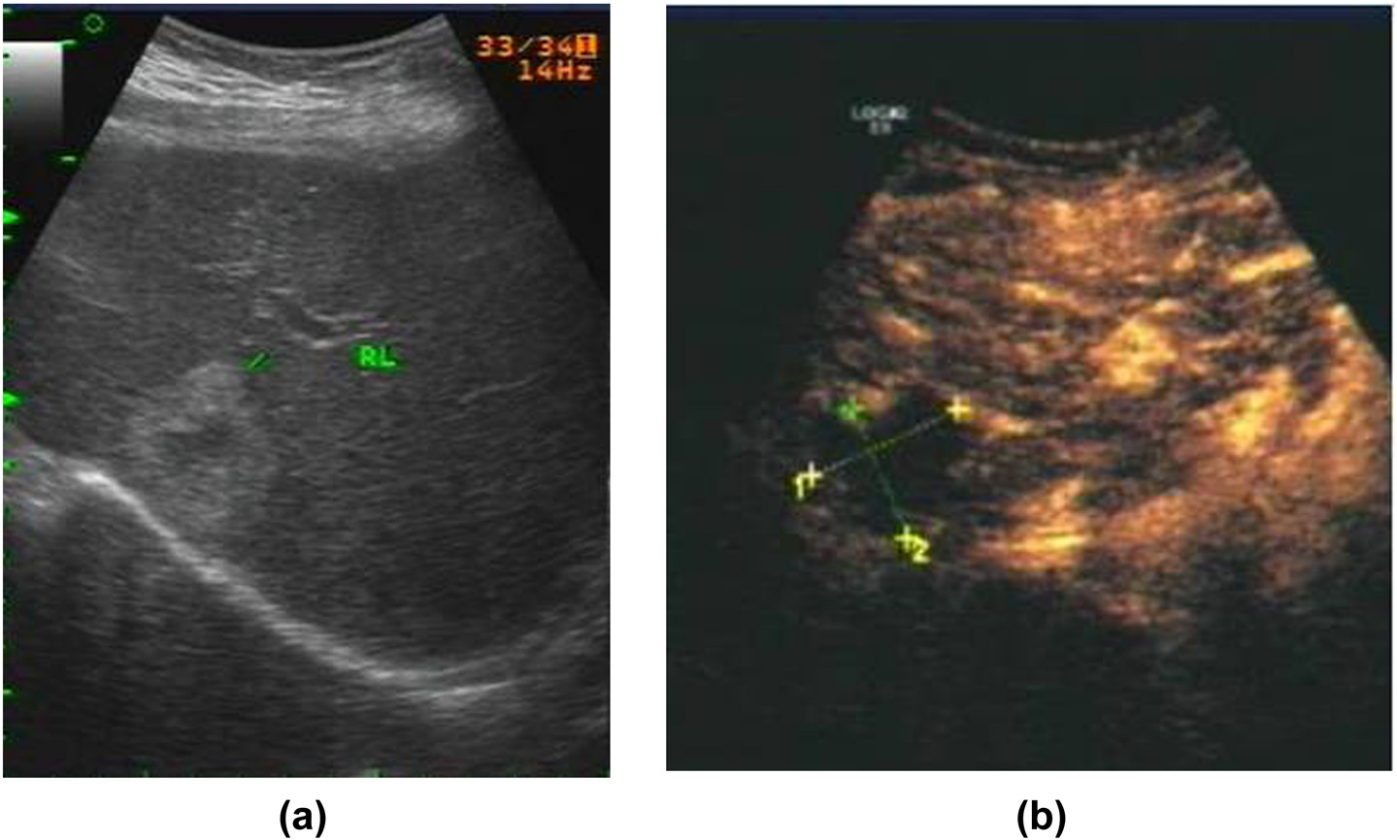
(a) Two-dimensional ultrasound imaging shows an irregular hyperechoic lesion in the right lobe of the liver with liquefaction in the center. (b) Contrast-enhanced ultrasound shows no contrast enhancement in any of the three phases.

**Figure 2:**
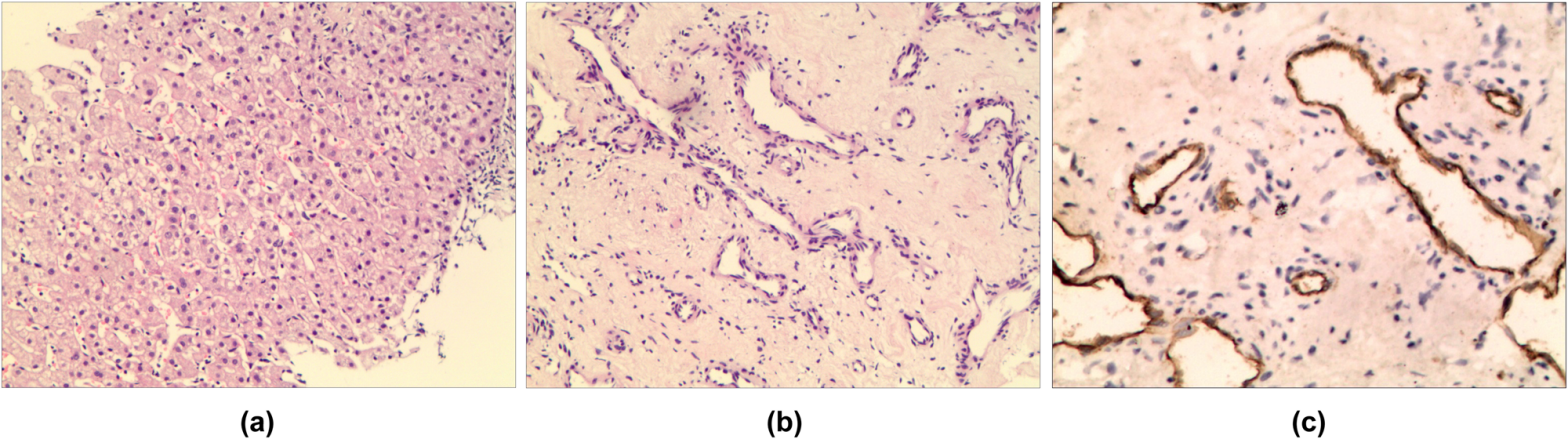
(a) ×100 and (b) ×200: the proliferation of interlobular fibrous tissue, small focal lymphocytic infiltration, and irregular vascular lymphatics can be seen between them. The endothelial cells are arranged in the shape of spikes. (c) ×200: CD31 (+++), CD34 (+++), FVIII–RAg (+), and Ki-67 <1%.

The patient had no history of hepatitis or cirrhosis and no history of alcohol consumption. She also denied any history of drug use or exposure to chemical poisons. Physical and experimental examination revealed nothing of note other than slightly elevated serum levels of alkaline phosphatase and γ-glutamyl transpeptidase. A contrast-enhanced ultrasound showed that there was no enhancement of the contrast medium at any time in the lesion, so it was considered to be focal necrosis with partial liquefaction (Figure 1). In order to further determine the nature of the lesion, an ultrasound-guided liver biopsy was performed. Pathological examination showed a proliferation of interlobular fibrous tissue and infiltration of small focal lymphocytes, in which there were vascular lymphatics of irregular size. The endothelial cells were arranged in the shape of a shoe nail. Immunohistochemistry with a result of Cluster of Differentiation 31 (CD31) (+++), CD34 (+++), Factor VIII–Related Antigen (FVIII−RAg) (+), and Ki-67 (antigen identified by monoclonal antibody Ki-67) <1% led to a pathological diagnosis of hepatic RH (Figure 2).

**Figure 3:**
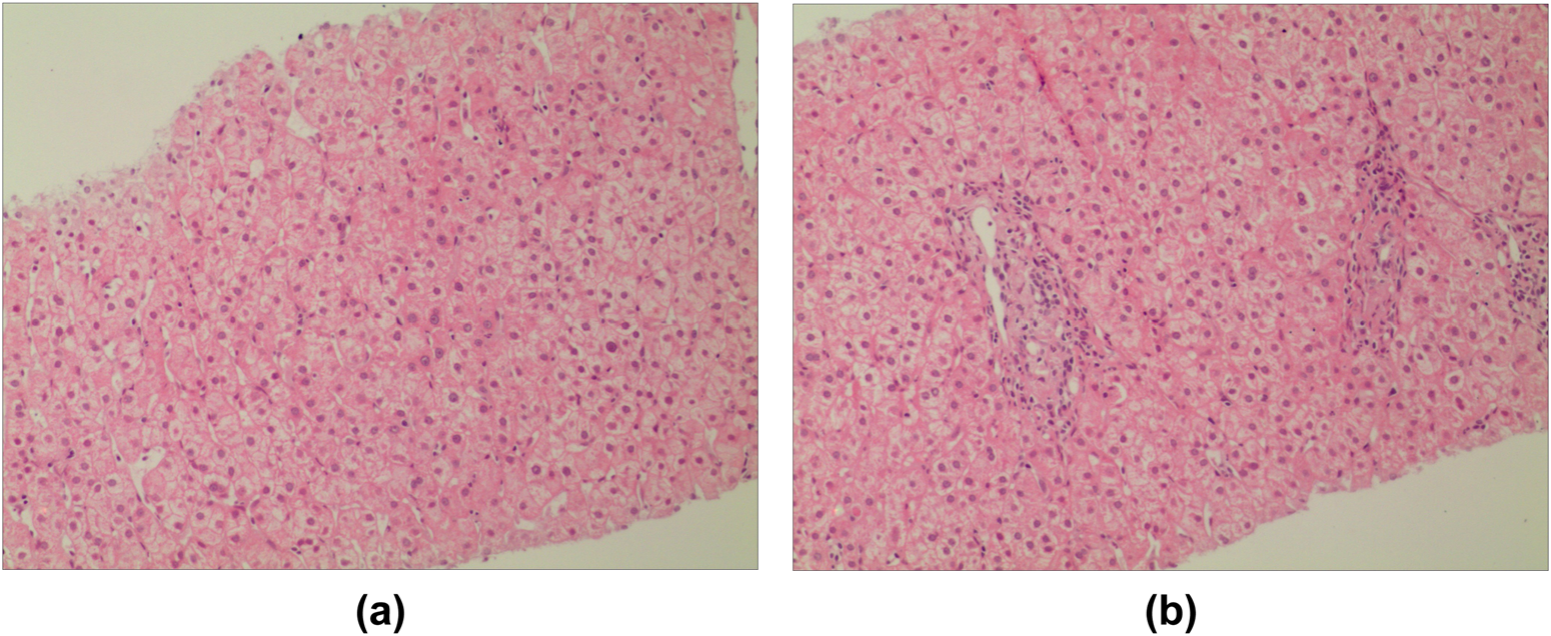
Infiltration of lymphocytes and monocytes in the portal area (×100).


After refusing surgical treatment, the patient underwent ultrasound-guided microwave ablation therapy and two supplementary treatments after 2 and 4 months. A biopsy was taken again after 6 months, and the pathological examination showed only an infiltration of focal lymphocytes and monocytes in the portal area (Figure 3). The patient has been followed up for 8 years with no tumor recurrence or metastasis.

**Informed consent:** Informed consent has been obtained from all individuals included in this study.
**Ethical approval:** The research related to human use has been complied with all the relevant national regulations, institutional policies, and in accordance with the tenets of the Helsinki Declaration and was approved by the Ethics Committee of The 940 Hospital of Joint Logistic Support Force of People’s Liberation Army.

## 3 Discussion

RH is a type of low-grade malignant angiosarcoma [3], and its causes are unknown. Although metastasis and malignancy are rare, RH is known to recur in approximately 50% of cases [4]. RH most commonly affects adult female patients and usually involves the skin and subcutaneous tissue of the lower extremities, but a few cases have been reported in the postauricular and gluteal regions, mandible, spleen, and jejunum [5,6,7,8,9]. The features of RH vary among patients, expressing as hyperhidrosis or erosion masses [4,10,11]. In the reported case, the lesions were surrounded by poor circumscription as a presentation of their histological character.

The main clinical manifestations of RH are single, slow-growing, and unclear local skin plaques. Its histopathology mainly manifests as unclear tumor tissue boundaries, the reticular distribution of blood vessels, a single layer of endothelial cells in the shape of shoe nails, and surrounding lymphocytes. In the present case, immunohistochemical staining showed that D2-40 (carcinoembryonic antigen M2A), CD31, CD34, and Ki-67 were positive [12].

The diagnosis of RH mainly relies on histopathological morphology and immunohistochemical markers. RH also needs to be differentiated from benign and malignant tumors with hobnail morphology, such as angiosarcoma and hobnail hemangioma. Angiosarcoma is a highly malignant tumor with high recurrence and mortality rates. Its pathological manifestations are vascular disorder, an irregularly shaped vascular cavity, poor differentiation of tumor cells, obvious heteromorphism, visible mitosis, and tumor cells of varying sizes with less cytoplasm, slight eosinophilia, light staining, and unclear boundaries. Hobnail hemangioma is a benign tumor often seen in children and teenagers that can be treated with surgical resection. Its pathological manifestation is expanding vessels, similar to a dermal tumor. In hobnail hemangioma, the papillary process can be seen in the lumen, the endothelial cells are shaped like protruding nails, an irregular and narrow vascular space can be seen in the deep layer, and it is surrounded by lymphocyte infiltration [13].

At present, the treatment of RH is a wide surgical excision with histopathologically confirmed tumor-free margins; long-term follow-up is essential. Radiotherapy, chemotherapy, pulsed dye laser therapy, and local corticosteroid injections have also been reported to be effective [14,15]. However, there have been no previous reports of the treatment of RH using ultrasound-guided microwave ablation. Microwave ablation is a local treatment method that uses the heat generated by high-frequency microwave electromagnetic energy to cause coagulation and necrosis of the pathological tissue, after which the necrotic tissue is absorbed by the body, thereby removing the local pathological tissue. This treatment has the advantages of minimal tissue damage and fast recovery [16]. It can be used to treat liver cancer, non-small-cell lung cancer, renal cancer, and other tumors [17]. For the treatment of small liver cancer, the five-year survival rate is similar to that of surgical resection, a radical operation.

The patient in the reported case was a middle-aged woman, and the final diagnosis was confirmed by pathology. As the patient refused to undergo surgery, she received ultrasound-guided microwave ablation. Two and four months after the first course of treatment, ultrasound-guided microwave ablation was performed again to ensure the complete necrosis of the tumor tissue. A biopsy was taken for pathological examination six months after the initial treatment, showing only an infiltration of focal lymphocytes and monocytes in the portal area. The patient has been followed up for eight years and is generally in good health, with no tumor recurrence or metastasis.

## 4 Conclusion

This was a case of hepatic RH exhibiting peculiar pathological features. Relevant imaging examinations, such as abdominal CT and magnetic resonance imaging, did not lead to a clear diagnosis, but an ultrasound-guided liver biopsy combined with immunohistochemistry confirmed the final diagnosis. This is the first reported case of hepatic RH and the only case of a patient with hepatic RH being treated successfully with ultrasound-guided microwave ablation. Surgical excision is still the first choice for the treatment of hepatic RH. As shown in this case, however, for patients who refuse to undergo surgery or have surgical contraindications, ultrasound-guided microwave ablation is an alternative treatment option. This case can enrich clinicians' understanding of hepatic angiosarcoma to a certain extent and can provide a reference for their next treatment options. However, this is only one case, and it still needs the support of evidence-based medicine to prove its effectiveness.
